# Antibacterial Activity of Phytochemicals Isolated from *Atractylodes japonica* against Methicillin-Resistant *Staphylococcus aureus*

**DOI:** 10.3390/molecules15107395

**Published:** 2010-10-21

**Authors:** Seung-Il Jeong, Seon-Young Kim, Sang-Jun Kim, Byung-Soon Hwang, Tae-Ho Kwon, Kang-Yeol Yu, Seung-Ho Hang, Koji Suzuki, Kang-Ju Kim

**Affiliations:** 1 Jeonju Biomaterials Institute, Jeonju 561-360, Korea; E-Mails: seungil@paran.com (S.-I.J.); seon02@jbmi.re.kr (S.-Y.K.); process95@jbmi.re.kr (S.-J.K.); hbs2015@nate.com (B.-S.H.); thkwon@jbmi.re.kr (T.-H.K.);kangyu@jbmi.re.kr (K.-Y.Y.); 2 Department of Laboratory Medicine, Veterans Hospital, Daejeon, 306-830, Korea; E-Mail: microhemato@hanmail.net (S.-H.H.); 3 GL Sciences Inc, Tokyo, Japan; E-Mail: ksuzuki@gls.co.jp (K.S.); 4 Department of Oral Microbiology, School of Dentistry and Biotechnology Institute, Wonkwang University, Iksan 570-749, Korea

**Keywords:** *Atractylodes japonica*, MRSA, MIC/MBC

## Abstract

Methicillin-resistant *Staphylococcus aureus* (MRSA) has been emerging worldwide as one of the most important problems in communities and hospitals. Therefore, new agents are needed to treat acute oral infections from MRSA. In this study, antibacterial compounds from the roots of *Atractylodes japonica* (*A*. *japonica*) were isolated and characterized. The compounds were isolated from the root extracts using HPLC-piloted activity-guided fractionations. Four *A*. *japonica* compounds were isolated and identified as atractylenolide III (**1**), atractylenolide I (**2**), diacetylatractylodiol [(6*E*,12*E*)-tetradeca-6,12-diene-8,10-diyne-1,3-diol diacetate, TDEYA, **3**). and (6*E*,12*E*)-tetradecadiene-8,10-diyne-1,3-diol (TDEA, **4**), which was obtained by hydrolysis of TDEYA. The minimum inhibitory concentrations (MICs) was determined in the setting of clinical MRSA isolates. Compound **4** showed anti-MRSA activity with a MIC value of 4-32 μg/mL. The overall results provide promising baseline information for the potential use of the extract of *A*. *japonica* as well as some of the isolated compounds in the treatment of bacterial infections.

## 1. Introduction

Methicillin-resistant *Staphylococcus aureus* (MRSA) accounts for a large proportion of hospital-acquired infections and is considered a serious problem because of its multi-drug resistant properties. Currently, vancomycin and its analog teicoplanin are the most effective antibiotics for MRSA infections, but their clinical use often results in unexpected side effects and the development of vancomycin-resistant *S. aureus* infections. The search for better drugs to combat MRSA infection is urgently needed.

*Atractylodes japonica* (Compositae) has traditionally been used for the treatment of water retention in the body. Administration of the aqueous extract of *Atractylodes japonica* to humans causes diuresis and its alcohol extract also shows a diuretic effect in mice [1,2]. *Atractylodes japonica* is known to be effective for the control of pain and treatment of arthritis. It was reported that the *Atractylodes* family possesses anti-inflammatory activity [3], and modulates the intestinal immune system [4]. Atractylon, a major component, and its derivatives isolated from the rhizome were shown to have antihepatic properties [5]. The sequiterpenoid diacetyl atractylodiol and its derivatives were isolated from the non-polar fraction [2]. Recently, it was reported that *A. japonica* root extract protects osteoblastic MC3T3-E1 cells against hydrogen peroxide-induced inhibition of osteoblastic differentiation [6]. However, the antibacterial effects of *A. japonica* on MRSA has not been evaluated. In the course of our ongoing project on the detection of bioactive compound from medicinal plants, the CHCl_3_-soluble extract of roots of *A. japonica* was found to exhibit distinctive antibacterial activity.

## 2. Results and Discussion

**Figure 1:**
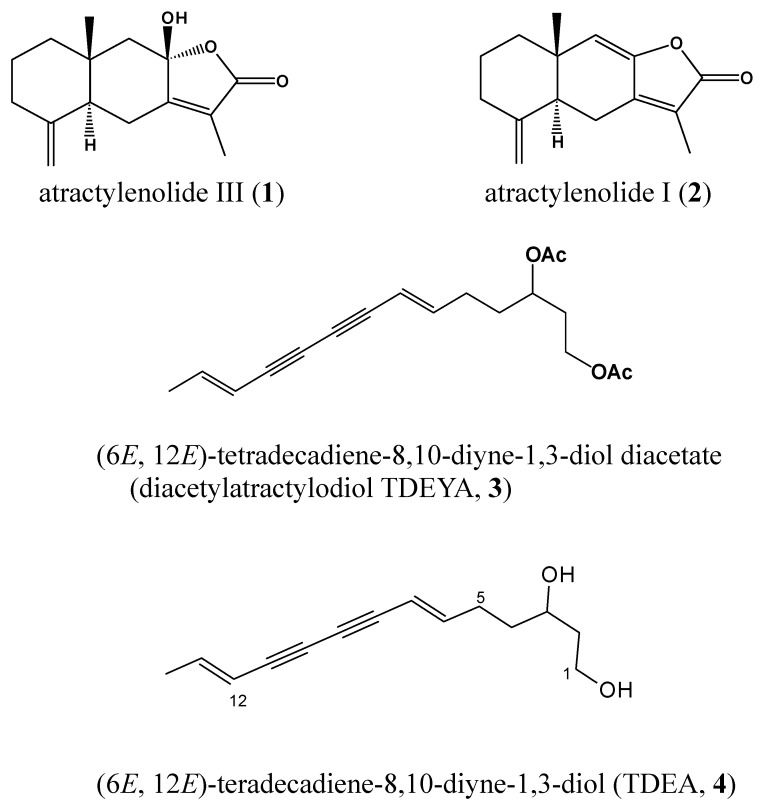
Chemical structures of isolated compounds from roots of *A. japonica*: atractylenolide III (1); atractylenolide I (**2**); (6*E*, 12*E*)-tetradecadiene-8,10-diyne-1,3-diol diacetate (diacetylatractylodiol TDEYA, **3**); (6*E*, 12*E*)-tetradecadiene-8,10-diyne-1,3-diol (TDEA, **4**).

Today, with the emergence of antibiotic-resistant pathogens like MRSA, a new approach to natural products must be taken. These natural products are more and more in demand due to their benefits without side effects. Therefore, our ongoing efforts to find bioactive natural products have led us to study the antibacterial activity of *A. japonica*, which has been known to possess a variety of pharmacological properties against arthritis, bronchitis and respiratory infectious disease and to contain more than 50 phytochemicals, including atractylon and its derivatives [7,8], sesquiterpenoids [9] and diacetyl atractylodiol including its derivatives [10,11]. To elucidate the antibacterial effects of *A. japonica*, the methanol extracts of *A. japonica* rhizome were fractionized with *n*-hexane, CHCl_3_, EtOAc, and *n*-BuOH. The fractions were tested for MIC determination using the microdilution broth method. The results were recorded as MIC values in Table 1. The CHCl_3_ fraction of *A. japonica* roots showed good antibacterial effects against two strains of *S. aureus*. The MIC of CHCl_3_ fraction shows equal efficacy to ampicillin at 32 μg/mL against ATCC 33591. This is an encouraging result in regards to the ability of MRSA to be resistant to most antibiotics. By further purifying the fractions, compounds **1**, **2** and **3** were isolated from the CHCl_3_ fraction and were confirmed to be atractylenolide III (**1**), atractylenolide I (**2**), and (6*E*,12*E*)-tetradeca-6,12-diene-8,10-diyne-1,3-diol diacetate (diacetylatractylodiol, TDEYA, **3**), respectively, by the comparison of their spectral data with those in the references [12]. Also, (6*E*,12*E*)-tetradecadiene-8,10-diyne-1,3-diol (TDEA, **4**) [11] was obtained by hydrolysis of TDEYA (**3**) (Figure 1).

The isolated compounds after were tested against different strains of *S. aureus* as shown in Table 2. Among the obtained isolates, TDEA (**4**) showed antibacterial activity with MICs ranging from 4 to 32 μg/mL independently.

**Figure 2:**
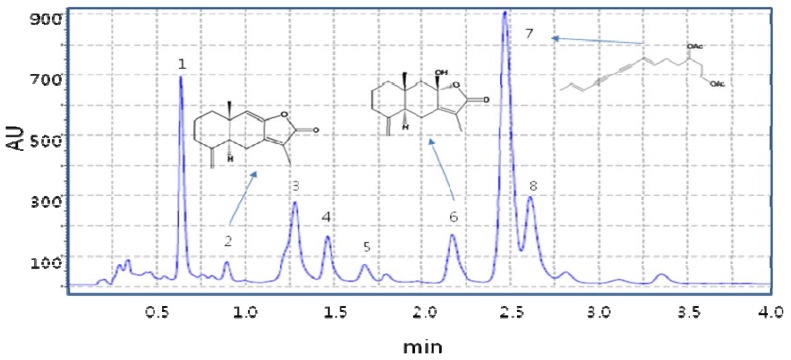
Smart HPLC chromatographic fingerprints of MeOH extract of *A. japonica*. The peaks marked with 2, 6 and 7 are atractylenolide III, atractylenolide I, (6*E*,12*E*)-teradecadiene-8,10-diyne-1,3-diol diacetate (diacetylatractylodiol, TDEYA), respectively. The separation conditions are described in the Smart HPLC chromatographic conditions.

Also, we investigated phytochemical profiles in *A. japonica* after separation using the developed Smart HPLC method. Typical chromatograms of the MeOH extract of *A. japonica* roots are shown in Figure 2. The identification of investigated compounds was carried out by comparison of their retention time and UV spectra with those obtained injecting standards in the same conditions, or spiking the samples with stock standard solutions. The developed Smart LC method indicated that the principle contained in the MeOH extract of *A. japonica* contained atractylenolide III (**1**), atractylenolide I (**2**), and (6*E*,12*E*)-tetradeca-6,12-diene-8,10-diyne-1,3-diol diacetate (diacetylatractylodiol, TDEYA, **3**), respectively. On Smart HPLC, (6*E*,12*E*)-tetradecadiene-8,10-diyne-1,3-diol (TDEA) was the main active component in the tested *A. japonica* that contributed to the antibacterial effect of MRSA. TDEA(4) was obtained by hydrolysis of TDEYA, which was not detected on HPLC in *A. japonica* extract.

Additionally, among the five different fractions, the chloroform fraction of *A*. *japonica* demonstrated a higher inhibitory activity against MRSA due to its bioactive constituents, in agreement with our Smart HPLC study.

The MIC values of the isolated compounds against MRSA are summarized in Table 2. Compounds **1**, **2**, and **3** showed inhibitory effect against *S. aureus* ATCC33591 (standard MRSA), *S. aureus* ATCC25923 (standard MSSA) as well as clinical isolates of MRSA, with MIC values of 8 to 128, 8 to 256 and 16 to 128 μg/mL, respectively. Compound **4** showed a significant MIC value (4 to 32 μg/mL) in comparison with the other compounds.

In conclusion, the isolated compounds are expected to be useful in the future for the study of anti-MRSA agents. However, for medicinal purposes, the safety and toxicity of these compounds need to be addressed. Also, further study is needed using vancomycin as a positive control for MRSA.

## 3. Experimental

### 3.1. Plant material

The fresh roots of *Atractylodes japonica* Koidz (Family Compositae) were collected in July 2009 at Jinan, Jeonbuk province, Korea. The plant was authenticated by Prof. Hong-Jun Kim, College of Oriental Medicine, Woosuk University. A voucher specimen (No. JSI 62) was deposited in the Herbarium of College of Oriental Medicine, Woosuk University.

### 3.2. Extraction and isolation of test material

Air-dried and powdered roots of *A. japonica* (3.0 kg) were extracted with two portions of MeOH (10 L) for one week at room temperature. To give 319g of dark-brown extract, which was suspended in water. The suspension was extracted with hexane, CHCl_3_, EtOAc and *n*-BuOH, respectively, and the organic layer was concentrated to dryness *in vacuo* to give the hexane (48 g), CHCl_3_ (62 g), EtOAc (23g), and *n*-BuOH fractions (61 g). The CHCl_3_ soluble portion (42 g) was chromatographed on silica gel (6 cm i.d. × 50 cm, 1.5 kg) eluted stepwise with a mixture of EtOAc and *n*-hexane (16:1→ 8:1→ 4:1 → 2:1 → 0:1, each 800 mL), yielding seven fractions (A1-A7). Fraction A1 was purified by prep-LC JAI gel W252 column; 100% MeOH; flow rate 3.5 mL/min; UV detection at 254 nm) to give **1** (15 mg). Fraction A3 was also applied to a prep-LC (JAI gel W252 column; 100% MeOH; flow rate 3.5 mL/min; UV detection at 254 nm) to give **2** (30 mg) and **3** (12 mg). These compounds were identified as atractylenolide III (**1**), atractylenolide I (**2**), and (6*E*,12*E*)-tetradeca-6,12-diene-8,10-diyne-1,3-diol diacetate (diacetyl-atractylodiol, TDEYA, **3**) [12]. Compound **4** ((6*E*,12*E*)-tetradecadiene-8,10-diyne-1,3-diol , TDEA) [11] was obtained by hydrolysis of **3** (see Figure 1).

### 3.3. Hydrolysis of TDEYA(3)

A solution of **3** (20 mg) in 3% K_2_CO_3_(cat.)/MeOH (7 mL) was heated at 60 °C for 10 min. After cooling, the solution was neutralized with 1 N HCl, and MeOH was extracted with Et_2_O and then washed with deionized water. The organic layer was dried over anhydrous Na_2_SO_4_ and evaporated *in vacuo* to obtain compound **4** as a pale yellow amorphous powder (8 mg).

### 3.4. Chromatographic fingerprint conditions

A LC800 series (Smart LC, GL sciences, Tokyo, Japan) system equipped with binary solvent delivery pump, auto sampler, degasser, system oven and UV-visible detector was used to achieve Smart LC fingerprints. The chromatographic separation was carried out on a 2.1 mm × 50 mm, 2 μm particle, Innertsil ODS-4 C18 column (GL sciences, Japan) maintained at 40 °C. The mobile phase consisting a mixture 0.05% aqueous phosphoric acid and acetonitrile in the ratio of 34:66 (*v*/*v*) with flow rate of 0.5 mL/min was employed. The detector wavelength was monitored at 254 nm. All injection volumes of sample and standards were 1.5 μL.

### 3.5. Antibiotics

All antibiotics, including ampicillin was purchased from Sigma Chemical Co. (St Louis, MO, USA).

### 3.6. Bacterial strains

The 12 MRSA isolates used in this study were clinical isolates from Wonkwang University Hospital, Iksan, Jeonbuk, Korea. The MRSA strains were defined on the basis of the occurrence of the *mecA* gene and of their resistance to ampicillin and oxacillin, according to the 2009 guidelines of the Clinical and Laboratory Standards Institute (CLSI) [16]. The standard strains of MRSA ATCC 33591 and *S. aureus* ATCC 25923, which is a methicillin susceptible *Staphylococcus aures* (MSSA), were used as control strains. After culturing all stains on Mueller-Hinton agar (MHA), all bacteria were resuspended in Mueller-Hinton broth (MHB) to give 10^8^ colony forming units/mL; the resuspended cells were then incubated.

### 3.7. Detection of mecA gene

Detection of the *mecA* gene in the MRSA strains was performed by polymerase chain reaction (PCR) amplication. For rapid extraction, one to five bacterial colonies were suspended in 300 L of cell lysis buffer and heated at 100 °C for 20 min. After centrifugation at 12,000 rpm for 10 min, 2 L of the supernatant was used for the DNA extraction. PCR reactions were performed using a MRSA Primer Mix Kit (Genotex Co., Korea). The PCR amplification consisted of 30 cycles (94 °C, 60 seconds; 55 °C, 60 seconds; 72 °C, 60 seconds). The primers used in this study were as follows: *mecA* forward primer, 5′-ATGAGATTAGGCATCGTTTC-3′; reverse primer, 5′-TGGATGACAGTACCTGAGCC-3′ [14]. The final PCR products were separated 0.2% agarose gel.

### 3.8. Minimum Inhibitory Concentration (MIC)

The standard agar dilution method was used to determine the MIC of the antibiotics alone in accordance with Clinical and Laboratory Standards Institute (CLSI) guidelines [16]. To determine the MIC of *A. japonica* extract and isolated compounds, the specimen was initially dissolved in minimal amount of dimethyl sulphoxide followed by water to give a range of concentrations from 0.325% to 5% with a volume of 2 mL. Each samples were diluted to give a final concentrations from 0.0625% to 1% in each agar plate. Cell suspensions (1 × 10^4^ colony-forming units/mL) of 12 MRSA isolates and standard strain (ATCC 25923 and ATCC 33591) were inoculated onto agar plates and then incubated at 35 °C for 18 h. The MICs were defined as the lowest concentration of a test compound that completely inhibited cell growth in visible.

## Figures and Tables

**Table 1:** Antimicrobial activity of *A. japonica* root MeOH extract and *n*-hexane, CHCl_3_, EtOAc, and *n*-BuOH fractions against *S. aureus* (ATCC 33591, ATCC 25923) strains.

*S. aureus* strain	MIC (μg/mL)					
	MeOH	Hexane	CHCl_3_	EtOAc	BuOH	Ampicillin
ATCC 33591	64	128	32	128	128	32
ATCC 25923	64	64	32	64	128	0.125

**Table 2:** The MICs/MBCs of *A. japonica* CHCl_3_ fraction (AJCH), atractylenolide III (**1**), atractylenolide I (**2**), (6*E*,12*E*)-tetradecadiene-8,10-diyne-1,3-diol diacetate (**3**) and (6*E*,12*E*)-tetradecadiene-8,10-diyne-1,3-diol (**4**), and ampicillin (AM) against *S. aureus* strains.

*S. aureus* strain	Class	*mec*A gene	MIC(μg/mL)					
			AJCH	1	2	3	4	AM
ATCC 25923	MSSA	-	32	64	64	32	16	0.12
ATCC 33591	MRSA	+	32	128	256	64	16	32
*Clinical isolates*								
*S. aureus* 78	MRSA	+	64	64	64	64	8	256
*S. aureus* M11	MRSA	+	64	64	64	16	8	256
*S. aureus* 21-8	MRSA	+	64	32	32	128	4	256
*S. aureus* 6-2	MRSA	+	128	64	64	64	8	256
*S. aureus* 7-3	MRSA	+	64	64	64	64	32	256
*S. aureus* 8-4	MRSA	+	64	128	128	64	16	256
*S. aureus* 9-5	MRSA	+	256	128	128	32	16	256
*S. aureus* 13-7	MRSA	+	32	32	32	32	4	128
*S. aureus* 27-9	MRSA	+	64	32	128	64	8	128
*S. aureus* 47-10	MRSA	+	32	32	32	64	16	512
*S. aureus* 105-13	MRSA	+	32	8	8	64	4	256
*S. aureus* 106-14	MRSA	+	32	16	16	32	4	128

(+) positive, (-) negative
